# Comparative study on fatigue evaluation of suspenders by introducing actual vehicle trajectory data

**DOI:** 10.1038/s41598-024-55873-1

**Published:** 2024-03-02

**Authors:** Yue Pan, Yiqing Dong, Dalei Wang, Sugong Cao, Airong Chen

**Affiliations:** 1 College of Civil Engineering, Tongji University, Shanghai, China; 2 School of Civil and Environmental Engineering, Nanyang Technological University, Singapore, Singapore; 3 Key Laboratory of Performance Evolution and Control for Engineering Structures (Ministry of Education), Tongji University, Shanghai, China; 4 Key Laboratory of Road and Bridge Detection and Maintenance Technology of Zhejiang Province, Hangzhou, China

**Keywords:** Civil engineering, Electrical and electronic engineering, Civil engineering, Electrical and electronic engineering

## Abstract

Suspenders play a crucial role in transmitting loads from the bridge deck to the main cable in a suspension bridge. They are susceptible to fatigue due to repeated dynamic loads, particularly traffic loads. Traffic Load Models (TLMs), typically created using Monte–Carlo simulation and Weigh-In-Motion (WIM) data, are employed to evaluate this fatigue. However, these models often overlook practical vehicle trajectories and spatio-temporal distribution, which compromises the precision of fatigue assessments. In this study, we introduce a novel 2D Intelligent Driver Model (2D-IDM) that incorporates actual vehicle trajectories, with a particular focus on transverse vehicle movement. This enhancement aims to improve the fidelity of existing TLMs. To provide a clear, qualitative, and quantitative understanding of the effects of fatigue evaluation with or without actual trajectory characteristics, we have structured this paper as a comparative study. We compare our proposed model, denoted as TLM *S-3*, with two observation-based models (*O-1* and *O-2*) and two simulation-based models (*S-1* and *S-2*). We conducted an experimental case study on a long-span suspension bridge, where the actual traffic load trajectory was obtained using a WIM-Vision integrated system. To calculate fatigue damage considering both longitudinal and transverse directions, we established a multi-scale Finite Element Model (FEM) using solid element types to simulate the bridge girder. This model can generate the stress influence surface of the bridge and has been verified in both static and dynamic aspects. Our detailed comparative analysis demonstrates the consistency of the proposed 2D-IDM with the actual measured traffic load trajectories. This indicates that our approach can enhance the fidelity and precision of fatigue evaluations for bridge suspenders.

## Introduction

Suspenders play a crucial role in suspension bridges, facilitating the transfer of loads from the bridge deck to the main cable. However, they are susceptible to fatigue issues and potential fractures due to repeated dynamic loads, as discussed in previous studies^1,2^. Therefore, evaluating the fatigue of each suspender is essential for the long-term operation of a large suspension bridge.

Given that traffic load is widely recognized as the primary contributor to the fatigue damage experienced by bridge suspenders^1,3^, on-site vehicle measurement systems, including weigh-in-motion (WIM) and surveillance systems, have become increasingly prevalent for long-span suspension bridges. Subsequently, traffic load models (TLMs) can be developed to evaluate the fatigue condition of suspension bridges by employing damage accumulation methods.

The WIM system primarily captures essential data points for each passing vehicle, including the time-point, lane position, and weight information^4^. On a macro scale, it compiles statistics on the number, dimensions, and distribution of vehicles, which can then be used to create TLMs for evaluating the performance of bridge suspenders.

There are two common approaches to traffic load modeling. Firstly, models can be directly generated using on-site WIM data. This involves inferring the travel history of each vehicle based on arrival time and speed, leading to the creation of traffic load distribution^5^. By applying spatial-temporal vehicle loading to the computational model, suspender fatigue damages can be calculated during structural monitoring. To enhance calculation efficiency for traffic loading, a standard fatigue vehicle is defined and employed for fatigue assessment^6^. Additionally, the analysis of vehicle-bridge interaction is considered to ensure precise calculation of dynamic responses^7^.

Traffic simulation is commonly used for modeling traffic loads when evaluating the fatigue of suspenders^8–10^ due to the need for data extrapolation and quick modeling, even when complete load information isn’t available all the time. For a long-span suspension bridge, a random traffic load simulator is specifically developed based on collected WIM data. The fatigue performance of the suspenders is assessed using Monte–Carlo sampling^11^. Additionally, microscopic traffic simulation methods have been introduced to enhance traditional TLMs^12,13^. These models can simulate individual traveling vehicles, providing a more accurate representation of the randomness and complexity of traffic on a long-span bridge.

However, WIM sensors have limitations as they only capture vehicles when they pass over the 4-m-long sensing area. While they provide detailed weighing information, they cannot capture the randomness and complexity of vehicle travel patterns. In recent years, newly developed techniques, especially the widely used machine vision methods, have shown promise as supplements to enhance the fidelity of TLMs.

Machine vision techniques, known for their capacity to monitor large areas, have gained widespread attention and application in bridge engineering^14^. They are particularly valuable in load monitoring^15,16^, displacement measurement^17,18^, and structural damage identification^19,20^. In load monitoring, these techniques enable the identification and recording of spatial-temporal vehicle information on the bridge deck^21^.

In recent years, deep learning algorithms have emerged as the leading approach for vehicle detection, owing to their end-to-end capabilities, robustness, and efficiency^22^. Object detection models like Faster-RCNN, SSD, and YOLO-series models have been employed to identify vehicles on roads and bridge decks^23–25^. These models have achieved a high identification precision, with a mean Average Precision of 0.977 and a high precision rate of 20.9 FPS^26^.

Furthermore, based on the identification of vehicles on the road, traffic characteristics can be extracted and categorized into three phases: (1) Traveling Features: Initially, the behaviors of vehicles are observed through practical traffic monitoring. Factors such as vehicle type, lane width, and traffic conditions influence vehicle travel preferences^27^. Vehicles tend to maintain relatively continuous movement within their lane, minimizing lateral shifts^28^. The time headways between vehicles are affected by vehicle types and lane positions, as confirmed by statistical tests^29^. (2) Vehicle-Following Models: Next, traffic flow on the road can be abstracted as a vehicle-following model. Following vehicles accelerate or decelerate based on their individual travel preferences and the behavior of the leading vehicle. This concept is commonly studied in models like the Intelligent Driver Model (IDM)^30^, Cellular Automata (CA)^31^, and others. (3) Simulation Enhancements: Moreover, recent years have seen precise enhancements in simulation methods. Artificial Neural Network models have been employed to simulate traffic dynamics in mixed traffic scenarios using real data^32^. Additionally, the concept of transverse position has been integrated into microscopic traffic simulations on bridge decks, building upon the Intelligent Driver Model (IDM)^33^.

In conclusion, the study of traffic identification and simulation has been a subject of extensive research over the years. While vehicles constitute the primary dynamic loads for long-span bridges, there has been limited exploration into how actual-measured vehicle trajectory features impact the creation of TLMs and, more critically, the assessment of fatigue damage to bridge suspenders. Therefore, there is a need for a comparative study that examines traffic load modeling with and without the inclusion of driving features to facilitate further discussion on this topic.

The aim of this study is to examine the characteristics of real-world vehicle trajectories and develop an improved TLM that takes into account both longitudinal and transverse vehicle movements, offering a more accurate fatigue evaluation of suspenders. To achieve this goal, we conducted an experimental case study on a long-span suspension bridge, allowing us to collect spatial-temporal traffic load data. We will then utilize this measured data to fine-tune the model’s parameters. Subsequently, we will conduct a detailed comparison of the traffic load characteristics and fatigue damage results using these refined TLMs. This comparative analysis presented in our paper will provide a thorough and quantifiable evaluation of fatigue based on the data gathered during our on-site experiments.

## Traffic load models for fatigue evaluation

**Figure 1:**
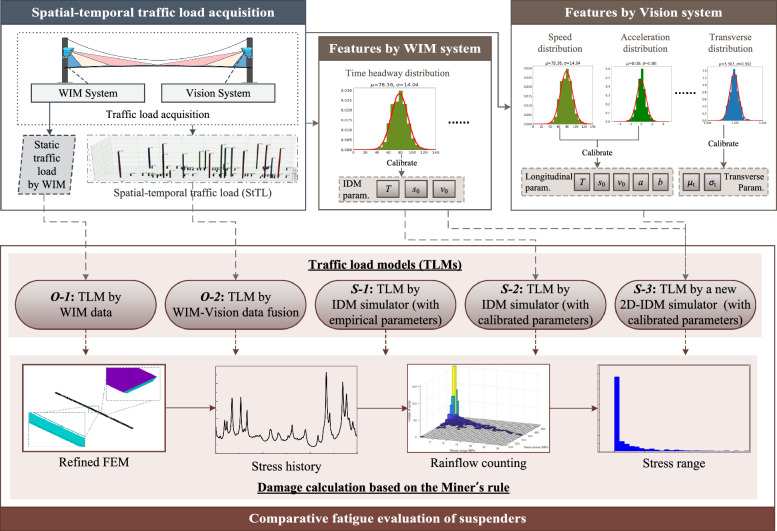
Comparative study on five TLMs for fatigue evaluation of bridge suspenders.

In this section, we delve into the impact of real-world vehicle trajectory characteristics on traffic load modeling. We establish two observation-based TLMs and three simulation-based TLMs. Notably, we utilize the Intelligent Driver Model (IDM) as our microscopic traffic simulator due to its ease of understanding and efficiency. Furthermore, we enhance the original IDM and introduce the 2D-IDM simulator to incorporate vehicle trajectory features into TLM. Figure 1 provides an overview of the comparative models and methods employed in this study.

To begin, vehicle load data was collected based on our previous study^34^. Vehicle load characteristics from both the WIM system and the Vision system are analyzed to create the five TLMs. Subsequently, the mechanical components of the girder are refined to generate a 3D finite element model of the bridge. Miner’s rule is then applied to calculate fatigue damages for the suspenders, enabling a comparison between the different TLMs.

### *O-1*: TLM by WIM data

The WIM system sensors record vehicle loads and speeds at a specific point. To extrapolate the vehicle distribution across the entire bridge deck, TLM *O-1* assumes that vehicles travel at a constant speed in the center of their lane. This model aligns with the approach used in prior studies^5^.

### *O-2*: TLM by WIM-vision data fusion

A vision system equipped with object detection algorithms can effectively identify vehicles on the bridge in real time. To acquire the actual vehicle trajectories, which represent the genuine vehicle distribution on the bridge deck, we utilize the WIM-Vision data fusion approach^25,26,34^.

TLM *O-2* is established as the practical spatial–temporal vehicle load distribution based on the fused data. Given that this result accurately represents the real positions and speeds of the vehicle loads, it serves as the benchmark for evaluating fatigue in bridge components.

### *S-1*: TLM by IDM simulator (with empirical parameters)

The Intelligent Driver Model (IDM), introduced by Treiber and Hennecke^35^, is among the widely recognized models for simulating microscopic traffic flow. IDM is a one-dimensional simulator that focuses on vehicle-following behavior.

**Figure 2:**
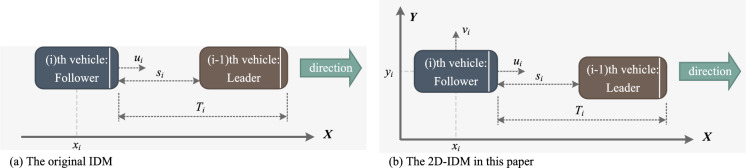
Illustration of the original IDM and the proposed 2D-IDM.

In IDM, each vehicle is expected to travel at its desired speed and adjust its acceleration or deceleration based on the behavior of the leading vehicle. The longitudinal motion of the follower vehicle (i.e., the i-th vehicle) is determined by Eq. (1) and is illustrated in Fig. 2a.

1 $$\begin{aligned} \begin{aligned}{}&\frac{du_i}{dt} = a_i \left( 1 - \left( \frac{u_i}{u_{0,i}}\right) ^\delta - \left( \frac{s^* \left( u_i, \Delta u_i \right) }{s_i}\right) ^2 \right) , \\&s^* \left( u_i, \Delta u_i \right) = s_{0,i} + u_i T_i + \frac{u_i \Delta u_i}{\sqrt{2 a_i b_i}}, \end{aligned} \end{aligned}$$

where $u_i$ and $\frac{du_i}{dt}$ are the vehicle speed and acceleration in the longitudinal direction, respectively. $\delta$ is the acceleration factor ($\delta = 4$ in this paper). $a_i$ and $b_i$ are the desired acceleration and comfortable deceleration values, separately. $u_{0,i}$ is the desired speed. $T_i$ is the safe time headway. $s_{0,i}$ is the minimum distance. Then to update the speed and position of the follower vehicle, Eq. (2) is utilized, where $\Delta t$ is the length of the updating step for the model.

2 $$\begin{aligned} \begin{aligned}{}&u_i(t+\Delta t) = u_i(t) + \frac{du_i}{dt} \cdot \Delta t, \\&x_i(t+\Delta t) = x_i(t) + u_i(t) \cdot \Delta t + \frac{1}{2} \cdot \frac{du_i}{dt} \cdot (\Delta t)^2. \end{aligned} \end{aligned}$$

TLM *S-1* primarily utilizes the IDM simulator with its empirical parameters in transportation simulation^36^, which are detailed in Table 1. Initially, each vehicle is generated using a Monte–Carlo (MC) approach based on WIM data^37–39^, including weight and axle information. The vehicle generation process is consistent for TLM *S-2* and *S-3*.

**Table 1:** Empirical parameters of the original IDM simulator.

$v_0\, (\text {km/h})$	$T \,(\text{s})$	$a\, (\text {m/s}^2)$	$b\, (\text {m/s}^2)$	$s_0 \,(\text{m})$
120	1.6	1.0	1.5	2.0

### *S-2*: TLM by IDM simulator (with calibrated parameters)

The fatigue of components is affected by the distribution of vehicles on the bridge deck, as noted in previous studies^40^. In Eq. (1), the variables $v_0$, *T*, and $s_0$ have an impact on vehicle speeds and distances, consequently influencing the load distribution across the entire bridge deck. Therefore, we introduce TLM *S-2*, which involves calibrating the parameters of the IDM simulator using WIM data.

### *S-3*: TLM by a new 2D-IDM simulator (with calibrated parameters)

In practical scenarios, vehicles on the bridge deck do not strictly adhere to the center of their lanes. As a result, the precise transverse positions are not accounted for in the original IDM. In this study, we introduce a new 2D-IDM simulator, as depicted in Fig. 2b, primarily to incorporate the transverse motion of each vehicle.

In the transverse direction, a vehicle’s movement reflects its lane-keeping behavior and exhibits random characteristics, as observed in previous studies^27,41^. The vehicle’s transverse movement within the lane can be likened to a random walk process. Consequently, the transverse movement of vehicles can be modeled and computed using Eq. (3).

3 $$\begin{aligned} &y_i (t+\Delta t) = y_i (t) + \Delta y \\&\Delta y \sim \mathcal {N}(0, \sigma _t^2) \\&y_{0,i} = \mu _t, \end{aligned}$$

where $y_i$ represents the relative transverse location, normalized with respect to the lane width. $\Delta t$ is the length of the model’s updating step. $\mu _t$ and $\sigma _t$ are the drift rate and volatility of the random walk process^42^, respectively. These two parameters require calibration based on observed data.

Hereby, TLM *S-3* is the proposed 2D-IDM simulator, where all parameters are calibrated using practical traffic load distribution data obtained from the observed WIM-vision fusion data.

## Bridge information and data acquisition

### Bridge information

**Figure 3:**
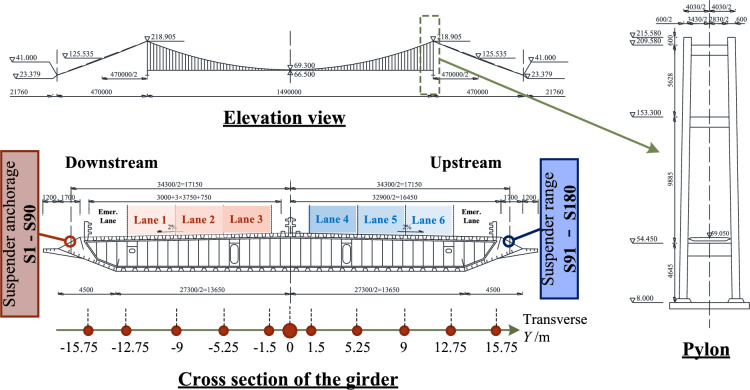
Main configurations of RSB.

To compare the effects of TLMs on suspender fatigue evaluation both qualitatively and quantitatively, an in-situ experiment is conducted on Runyang Suspension Bridge (RSB) on the Yangtze River in China. It is a long-span suspension bridge with a flat steel box girder and two H-shaped reinforced concrete pylons. The bridge has a central span of 1490 m, two side spans of 470 m, and a height of pylon 207 m. The overall layout of the bridge is shown in Fig. 3.

In addition, the spacing between two neighboring suspenders in the longitudinal direction is mostly 16.1 m, and 20.5 m for the distance of suspenders closest to the pylons. There are overall 180 suspenders and 2 central buckles adopted to connect the main cable with the bridge deck. To be clear, the downstream suspenders are denoted as S1 to S90, and upstream as S91 to S180. Besides, the typical cross-section of the girder is 38.7 m in width and 3 m in height. Depicted in Fig. 3, six normal lanes and two emergency lanes are arranged above the deck, with widths of 3.75 m and 3 m, respectively.

### Instrumentation

**Figure 4:**
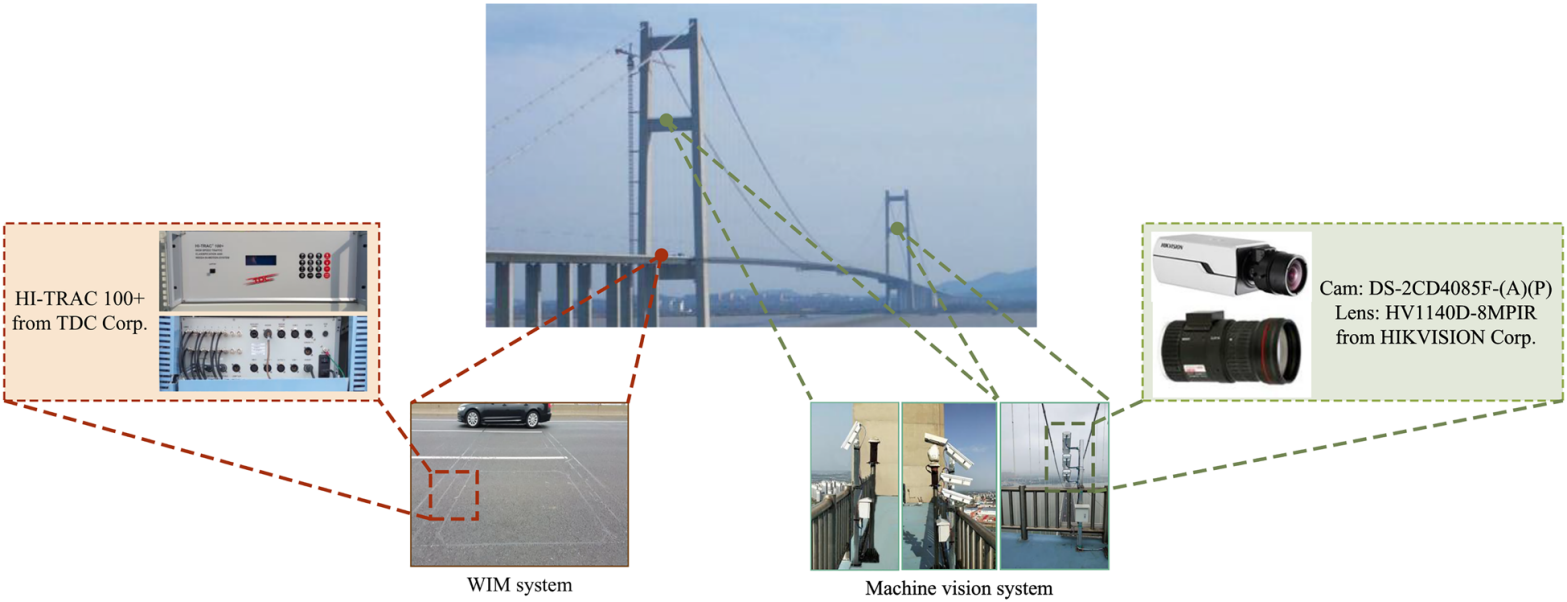
The in-situ instruments for full-bridge traffic load acquisition.

The instruments for full-bridge traffic load acquisition mainly consist of the WIM system and machine vision system, depicted in Fig. 4. The WIM system adopted commercial devices HI-TRAC 100+ from TDC Corp. While the machine vision system was self-developed, containing six cameras to cover the entire bridge deck area. The camera DS-2CD4085F-(A)(P) and lens HV1140D-8MPIR from HIKVISION Corp were selected.

**Figure 5:**
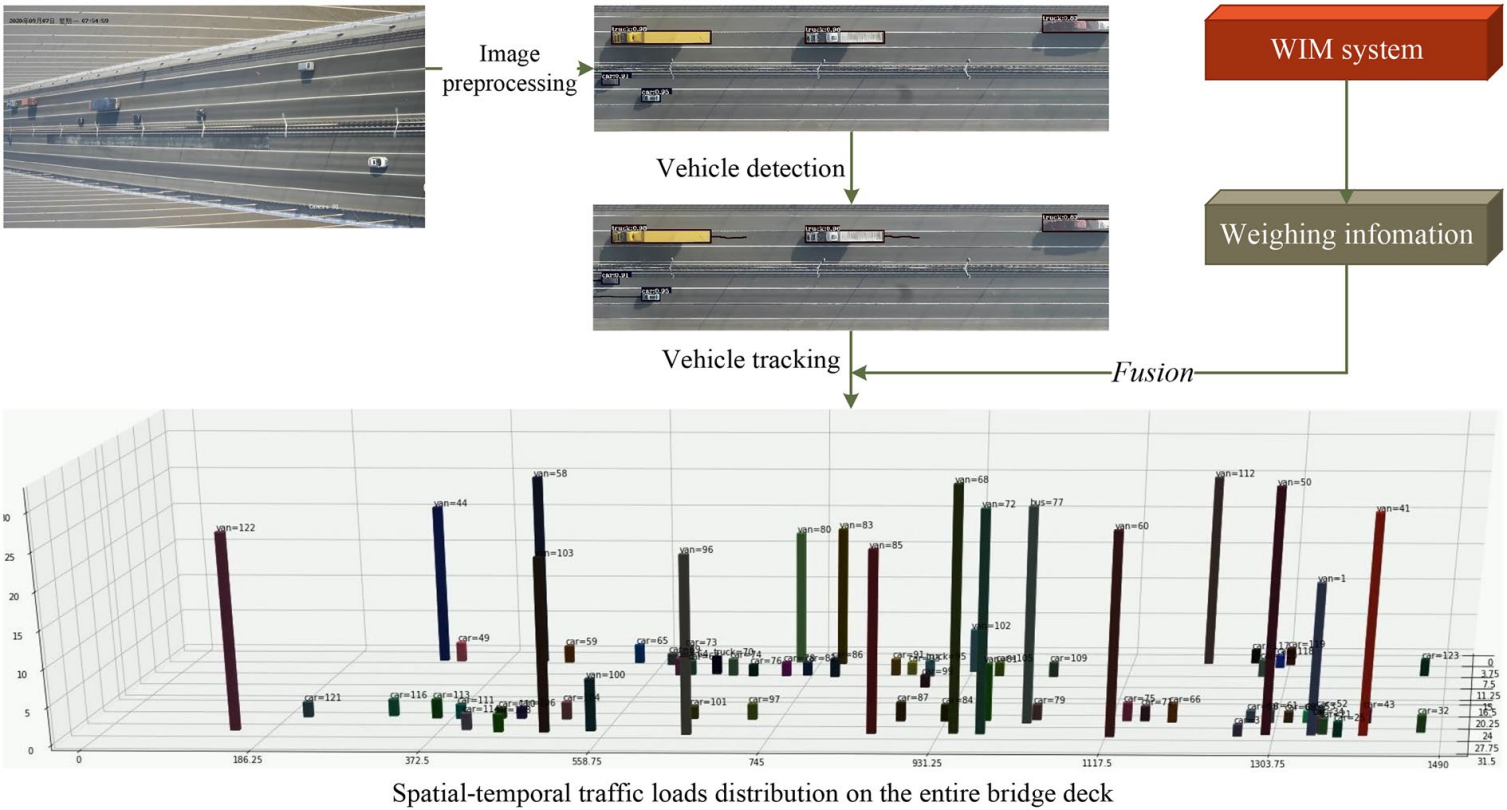
The procedure of traffic load acquisition.

Figure 5 illustrates the software and algorithms utilized by the machine vision system. The procedure commences with the preprocessing of each camera’s video frame, transforming it into a standardized top-view image. Following this, a deep learning-based vehicle detection model, specifically the You Only Look Once (YOLO) model, is trained and employed to identify vehicles within the frame. In our research, we achieved a high mean Average Precision of 96.2% following the optimization of hyper-parameters^34^. Upon completion of vehicle detection, the Vehicle Simple Online and Realtime Tracking (VehicleSORT) algorithm is activated for real-time tracking, facilitating the acquisition of vehicle trajectories. Our method attained a multi-object tracking precision of 99.28%. Subsequently, the WIM system gathers weighing data, which is then integrated with the trajectory data. This integration results in the spatial-temporal traffic load distribution across the entire bridge deck.

For a comprehensive understanding of the algorithms employed, our previous studies^34,43^ can be referred to.

## Fatigue damage evaluation of suspenders

In order to accurately evaluate the fatigue status of a suspender, it is essential to acquire stress curves under traffic loads, which requires the utilization of a computable finite element model (FEM) of the bridge. Subsequently, these stress curves are employed to apply the accumulative damage rule for calculating the fatigue life.

### FEM refining and verification

Typically, fatigue evaluation for bridge components relies on finite element models (FEM) that employ beam-type elements to represent the main girder. However, these models lack the ability to account for the transverse positions of vehicle loads. Therefore, in this study, we have improved the FEM for the experimented bridge by replacing the beam-type elements with shell-type elements to represent the bridge deck.

**Figure 6:**
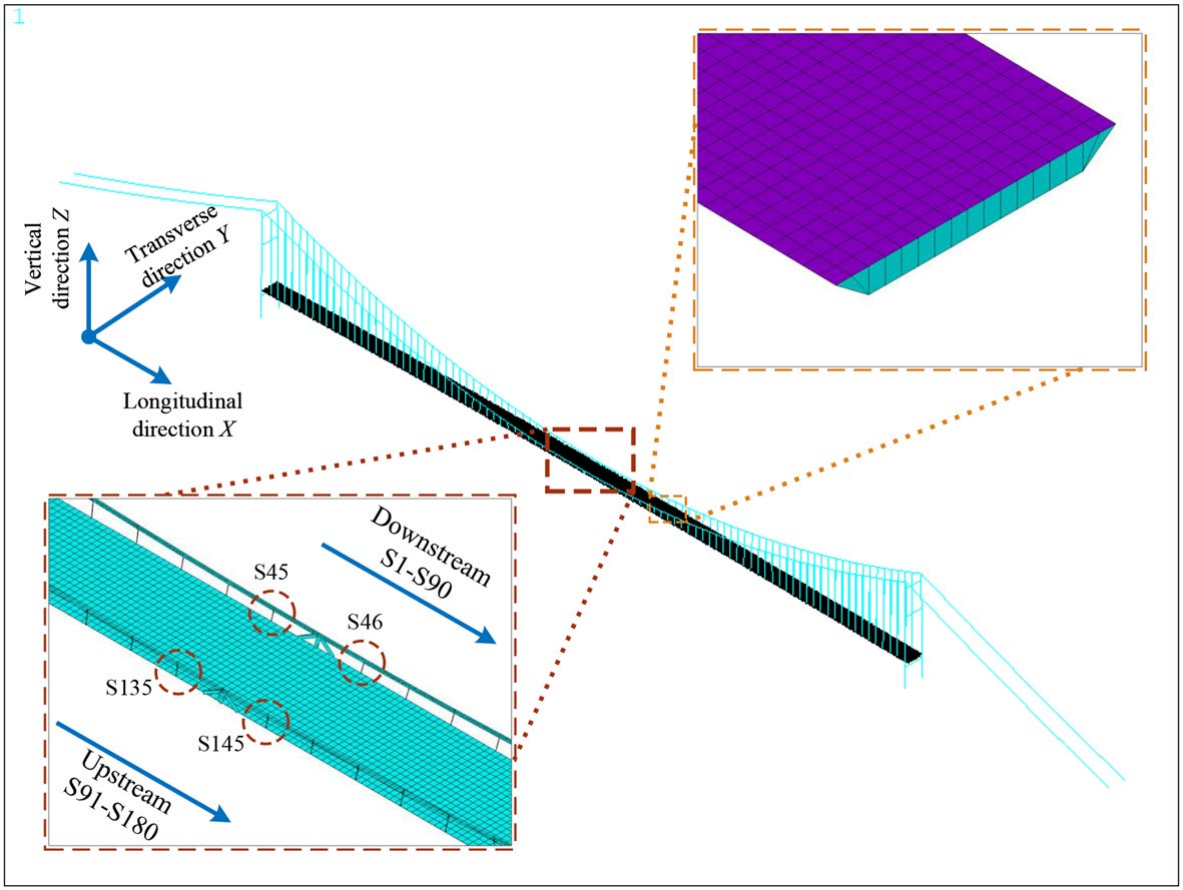
The refined FEM of RSB.

Specifically, we utilized the ANSYS software for modeling, as depicted in Fig. 6. The steel box girder is represented using the Shell63 element type, while the pylons and central buckles are modeled with the Beam4 element type. Additionally, the Link10 element type is employed to represent the main cables and suspenders.

**Figure 7:**
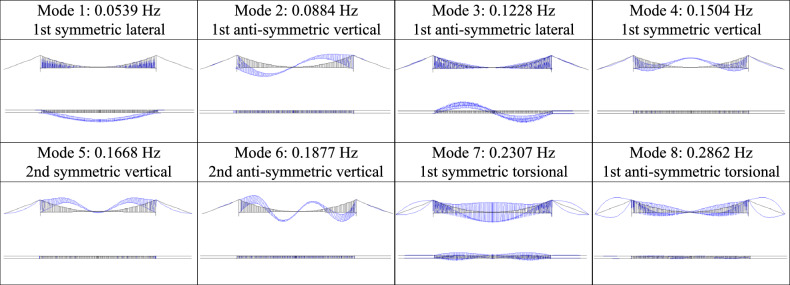
Mode shapes of RSB.

Next, the dynamic properties are computed to validate our model, and the outcomes are presented in Table 2. Mode shapes of RSB are also plotted in Fig. 7. In-situ testing results from a previous study^44^ are utilized as field-estimated frequencies for error assessment. The results indicate a close alignment between the modal parameters of the FEM and the measured values. This alignment encompasses both the characteristics of modes and their associated frequencies.

**Table 2:** Modal frequencies of RSB.

No.	Nature of mode	Field estimated (Hz)	FEM (Hz)	Error (%)
1	1st symmetric lateral	0.0586	0.0539	− 8.0
2	1st anti-symmetric vertical	0.0877	0.0884	0.8
3	1st anti-symmetric lateral	0.1221	0.1228	0.6
4	1st symmetric vertical	0.1587	0.1504	− 5.2
5	2nd symmetric vertical	0.1685	0.1668	− 1.0
6	2nd anti-symmetric vertical	0.1880	0.1877	− 0.2
7	1st symmetric torsional	0.2417	0.2307	− 4.6
8	1st anti-symmetric torsional	0.3077	0.2862	− 7.0

Short suspenders, such as S45, S46, S135, and S136 positioned at the mid-span, are more susceptible to experiencing higher stress amplitudes compared to their longer counterparts. This heightened vulnerability makes them more prone to fatigue-related issues^7,45^. As a result, our analysis in this paper focuses on these specific suspenders for representation.

**Figure 8:**
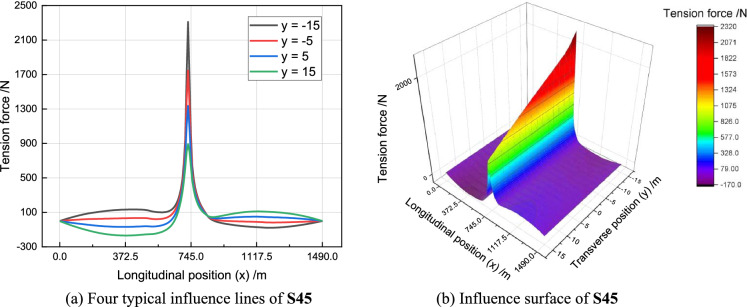
The influence lines and influence surface for tension force of S45.

To assess the impact of vehicle load on tension force, we consider the unit influence surface (UIS) for a suspender. This is achieved by applying a 10 kN (equivalent to 1t) concentrated load across the entire bridge deck, as illustrated in Fig. 8. Longitudinal and transverse directions of RSB are denoted as *x* and *y*, respectively. These designations are consistent with the FEM model depicted in Fig. 6. The influence surface curve reveals that the tension force induced by the vehicle load varies both longitudinally and transversely. Meanwhile, the tension force is significantly influenced by only a small region near the suspender’s anchorage.

### Fatigue evaluation based on Miner’s rule

Since the UIS does not account for the dynamic effects of vehicles, a dynamic amplification factor (DAF) need to be incorporated. The DAF is specified in both the Specifications for Design of Highway Bridges by the Ministry of Transport of the People’s Republic of China (MOTPRC)^46^ and the Load and Resistance Factor Design by the American Association of State Highways and Transportation Officials (AASHTO)^47^.

In this study, we employ the dynamic factor $\mu$ from MOTPRC^46^, which is determined based on the natural frequency *f* as shown in Eq. (4). Consequently, the stress in the suspender needs to be adjusted according to Eq. (5). In these equations, $T_d$ and $T_v$ represent the tension forces induced by dead load and vehicle load, respectively, while *A* denotes the area of the suspender.

4 $$\begin{aligned} \mu = {\left\{ \begin{array}{ll} \begin{array}{ll} 0.05 &{} when\ f < 1.5 {\text{ Hz}} \\ 0.1767\ln f - 0.0157 &{} when\ 1.5{\text{ Hz}} \le f \le 14{\text{ Hz}} \\ 0.45 &{} when\ f > 14{\text{ Hz}} \\ \end{array} \end{array}\right. }, \end{aligned}$$

5 $$\begin{aligned} \sigma = \frac{T_d + T_v \cdot \mu }{A}. \end{aligned}$$

After the time history of the suspender’s stress is obtained, the rainflow counting approach is used to statistically process the stress curve and determine the fatigue damage. The fatigue damage, denoted as *D*, is calculated according to the Miner rule or the linear damage accumulation rule, as defined in Eq. (6).

6 $$\begin{aligned} D = \sum \frac{n_i}{N_i} = \frac{n_1}{N_1} + \frac{n_2}{N_2} +\cdots + \frac{n_n}{N_n}, \end{aligned}$$

where $n_i$ is the number of stress ranges $\Delta \sigma _i$. $N_i$ denotes the fatigue life by stress range set as $\Delta \sigma _i$.

Based on previous studies^48,49^, the $S-N$ curve of steel wires in a suspender can be derived from Eq. (7). Here, *N* represents the maximum number of cycles a steel wire can endure under the stress range $\Delta \sigma$.

7 $$\begin{aligned} \log N = 13.95 - 3.5 \log \Delta \sigma . \end{aligned}$$

Simultaneously, the stress range’s initial history, obtained from the loading, needs to be adjusted by the suspender’s mean stress. The Goodman diagram is employed in this study to make this adjustment, as defined below:

8 $$\begin{aligned} \Delta \sigma _{rev} = k_{rev} \Delta \sigma , \end{aligned}$$

where $\Delta \sigma$ and $\Delta \sigma _{rev}$ represent the stress range before and after the revision, respectively; $k_{rev}$ is the coefficient of amplification defined as:

9 $$\begin{aligned} k_{rev} = \frac{1}{1 - \sigma _m/\sigma _b}, \end{aligned}$$

where $\sigma _m$ and $\sigma _b$ are the mean stress and ultimate tension strength, separately.

## Comparative results analysis

In this section, comparative results of the five defined TLMs are presented and discussed. Firstly, the traffic load history during the experiment was collected and analyzed to calibrate the parameters for the models. Subsequently, a detailed comparative study was conducted on traffic generation and fatigue evaluation of suspenders. Finally, the influence factors and error analysis were discussed.

### Parameter calibration of simulation-based TLMs

During the experiment, a total of 2673 vehicles were recorded by the systems. The Genetic Algorithm (GA)^50^, a proven effective and efficient method for parameter calibration, was employed to calibrate the five parameters in the longitudinal direction of IDM.

Specifically, the population size and maximum number of iterations were set to 200 and 300, respectively. Additionally, the mutation probability was set to 0.001. The calibration results of the longitudinal parameters of IDM, categorized by six distinct lane positions on the bridge, are presented in Table 3. The precision of the calibrated model was evaluated using the Mean Absolute Error (MAE), defined as Eq. (10). As demonstrated in Table 3, more precise parameters can be attained through detailed categorization, including lane categorization, as the MAE for each lane is lower than that when all lanes are calibrated together.

10 $$\begin{aligned} MAE = \frac{1}{N} \sum _{i=1}^N \left| \hat{u}_i - u_i \right| , \end{aligned}$$

where *N* is the amount of the vehicles. $\hat{u}_i$ and $u_i$ are the predicted and actual speed of the *i*-th vehicle, respectively.

**Table 3:** Calibration results of longitudinal parameters based on GA.

Lane No.	$v_0$ (m/s)	*T* (s)	*a* (m/s^2^)	*b* (m/s^2^)	$s_0$ (m)	MAE (m/s)
Lane 1	18.26	2.48	1.01	1.47	7.61	0.807
Lane 2	22.30	2.54	0.99	1.43	7.98	1.005
Lane 3	27.44	2.56	0.95	1.42	7.97	0.593
Lane 4	26.91	2.52	0.89	1.44	7.78	0.762
Lane 5	22.46	2.3	0.87	1.48	6.88	1.035
Lane 6	19.26	2.37	0.88	1.48	7.3	0.874
All lane	22.32	2.53	0.99	1.45	7.71	1.084

On the other hand, the transverse parameters of 2D-IDM in Eq. (3) are also calibrated, and the results for $\mu _t$ and $sigma_t$ in each lane are presented in Table 4.

**Table 4:** Calibration results of transverse parameters.

Lane No.	Lane 1	Lane 2	Lane 3	Lane 4	Lane 5	Lane 6
$\mu _t$	0.626	0.562	0.530	0.487	0.476	0.545
$\sigma _t$	0.00357	0.00307	0.00415	0.00413	0.00330	0.00333

**Figure 9:**
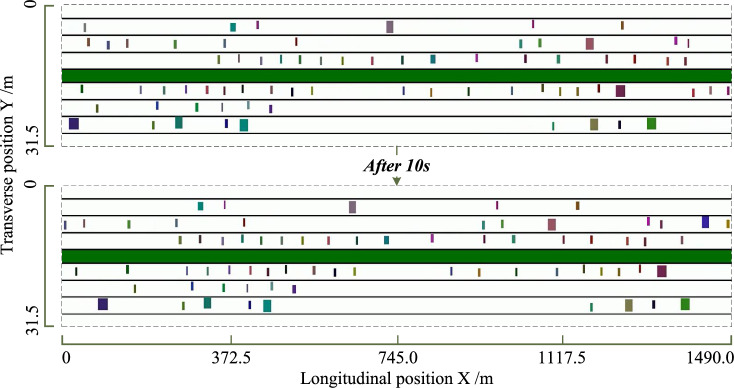
Two time-points of simulated full-bridge traffic loads by 2D-IDM based TLM *S-3*.

Dynamic traffic loads can be simulated after the calibration procedure. Specifically, for the 2D-IDM-based *S-3* model, two time-points of full-bridge traffic loads are depicted in Fig. 9. The proposed 2D-IDM not only retains the acceleration, deceleration, and car-following features of the original IDM, but also incorporates the stochastic transverse movements of each vehicle within its lane.

### Comparative analysis of traffic loads simulation

**Figure 10:**
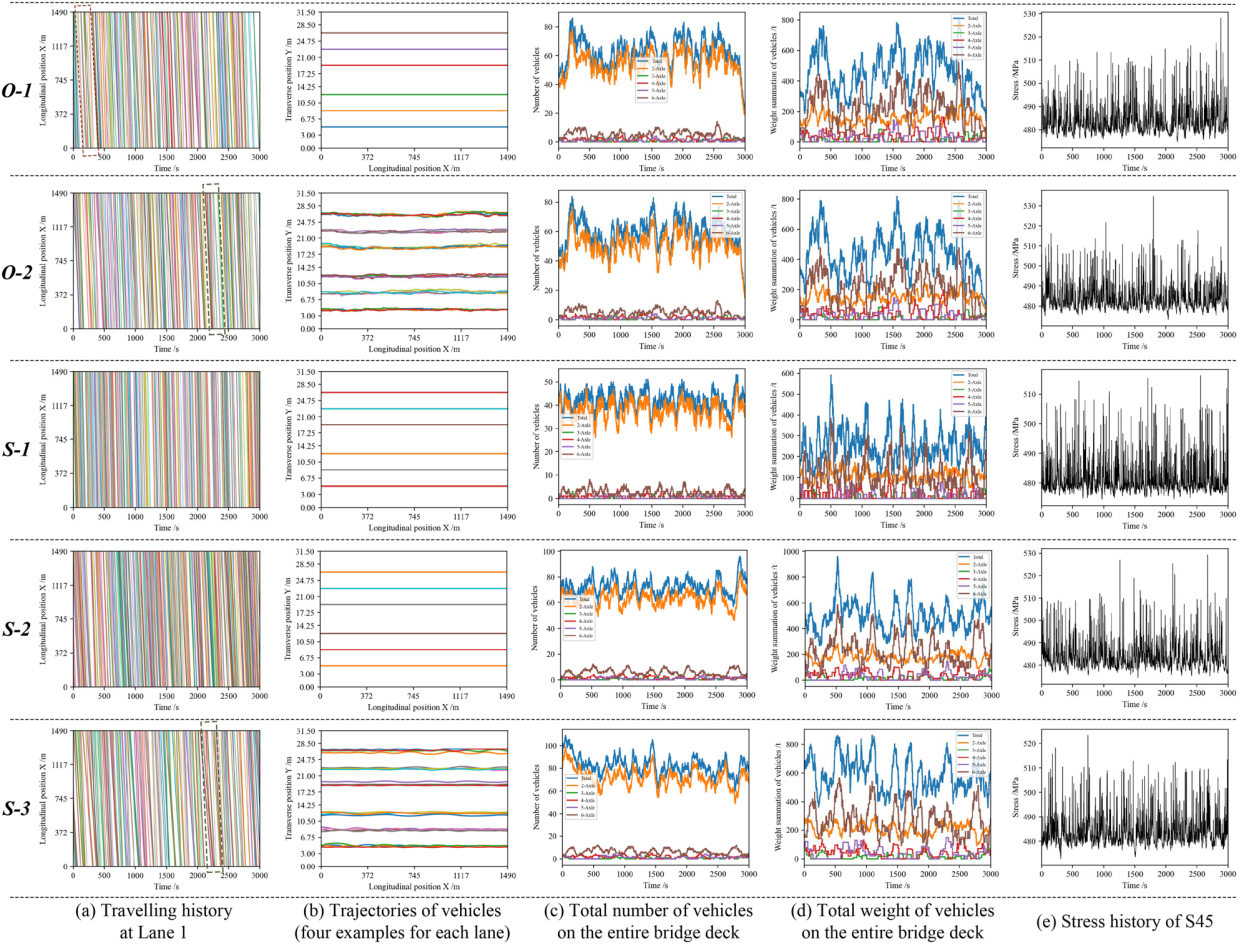
Comparative results of traffic load simulation from *O-1* to *S-3*.

Based on the measured data and calibrated parameters, the five TLMs are used to generate vehicles on the bridge deck. Data for one hour is produced for each model from *O-1* to *S-3*, and the resulting spatial-temporal traffic load is illustrated in Fig. 10.

In Fig. 10a, the traveling history of vehicles provided by each model at Lane 1 is plotted. The phenomenon of vehicle following and deceleration is observed in both *O-2* and *S-3*, as shown in the green-dashed rectangles. In contrast, vehicles in *S-1* and *S-2* only travel successively, and speed variations are barely observed. Additionally, in *O-1*, there are instances where the follower vehicle crossed over the leader vehicle (red-dashed rectangle) due to the model using speeds from the WIM system without considering topological relationships among vehicles.

Figure 10b depicts the trajectories of vehicles and highlights four examples for each lane. Transverse vehicle movements are observed in *O-2* and *S-3* but are not reflected in *O-1*, *S-1*, and *S-2*, which assume that the vehicles are traveling in the center of the lane.

Figure 10c,d illustrate the total number and weight of vehicles on the entire bridge deck, respectively. *O-1*, *O-2*, *S-2*, and *S-3* exhibit similar features in terms of vehicle counts and compositions. However, because the parameters, especially the speeds, are not calibrated, the number of vehicles on the deck in *S-1* is much lower than in the other TLMs.

### Comparative analysis of fatigue evaluation of suspenders

**Figure 11:**
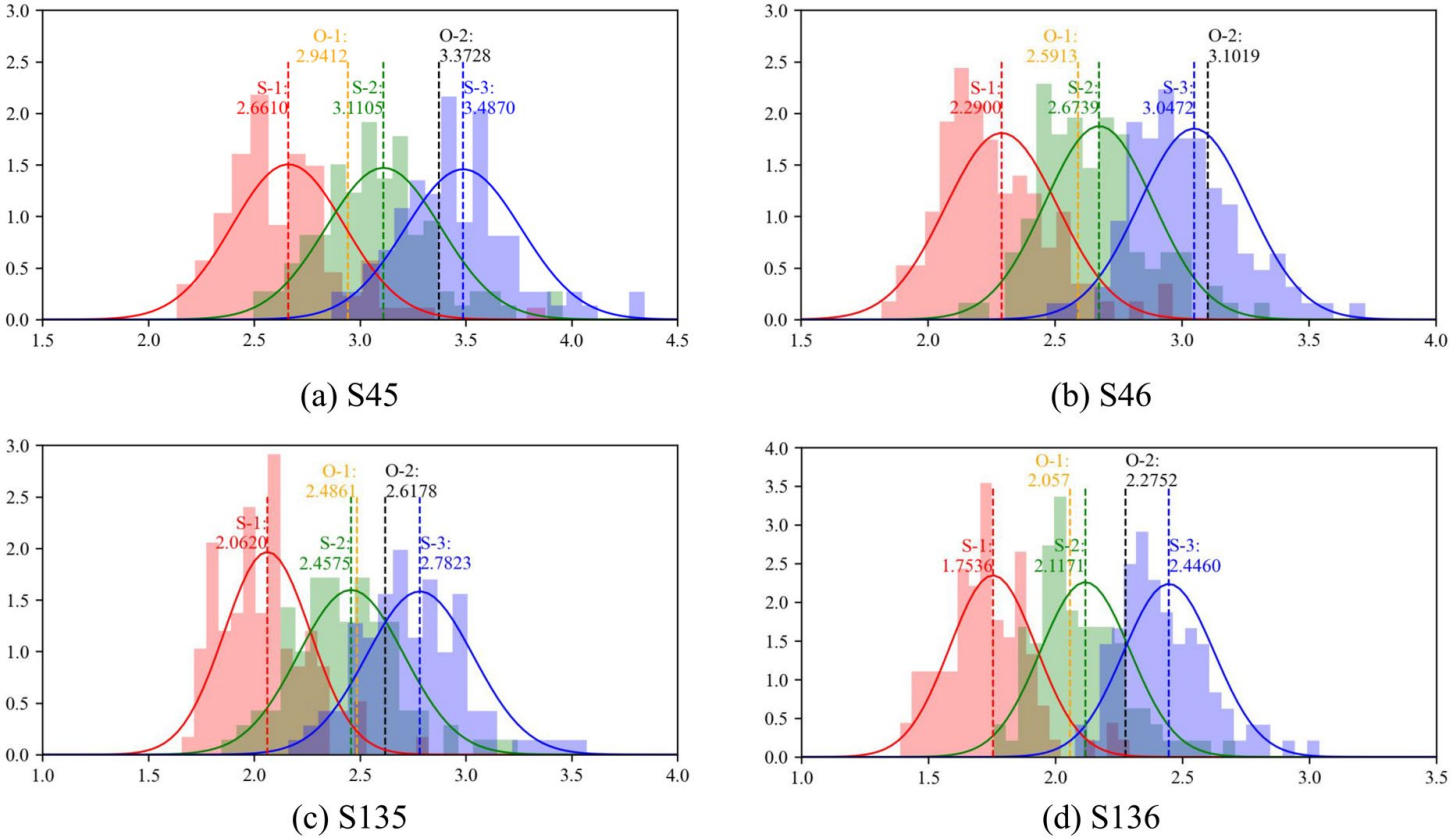
Comparative fatigue damage from *O-1* to *S-3*.

The fatigue damages of the RSB’s short suspenders, namely S45, S46, S135, and S136, are calculated using the five TLMs *O-1* to *S-3*. For *S-1*, *S-2*, and *S-3*, hundreds of trials are conducted to obtain the damage distribution of these simulation models. The results for fatigue damage are presented in Fig. 11, and a comparison of quantitative damage disparities is provided in Table 5.

The fatigue damage distributions in TLMs are influenced by the number of random simulations, as observed in Fig. 11. *O-1* and *O-2* are deterministic models, resulting in single values. Based on the normal distribution curves generated from histograms, the deviations of *S-1*, *S-2*, and *S-3* are consistent. However, the values of *S-3* tend to align more closely with the benchmarking model *O-2* than the other two simulation models. This suggests that *S-3* is likely to provide fatigue results that closely resemble the benchmarking model *O-2* for each suspender.

Table 5 presents quantitative results for comparison, with *O-2* serving as the benchmark model for evaluating disparities since its traffic load distribution is obtained through practical measurements. In general, the 2D-IDM-based TLM *S-3* exhibits less disparity with *O-2*, featuring a 4.74% positive average error, suggesting it may provide an additional margin of safety. On the other hand, *S-1*, which is not calibrated with observed data, demonstrates poorer damage results and consequently greater disparity than the other models. Moreover, *O-1* and *S-2* are models based solely on data from the in-situ WIM system. While not as precise as *S-3*, their average errors, averaging 10.97% and 8.66% respectively, are still acceptable for engineering applications with fewer constraints.

**Table 5:** Comparative results of fatigue damage from *O-1* to *S-3*.

Suspender no.	*O-1*/$\times$10E−8	*O-2* (Benchmark)/$\times$10E−8	*S-1*/$\times$10E−8	*S-2*/$\times$10E−8	*S-3*/$\times$10E−8
S45	2.9412/− 12.80%	3.3728/0.00%	2.6610/− 21.10%	3.1105/− 7.78%	3.4870/3.39%
S46	2.5913/− 16.46%	3.1019/0.00%	2.2900/− 26.17%	2.6739/− 13.80%	3.0472/− 1.76%
S135	2.4861/− 5.03%	2.6178/0.00%	2.0620/− 21.23%	2.4575/− 6.12%	2.7823/6.28%
S136	2.0570/− 9.59%	2.2752/0.00%	1.7536/− 22.93%	2.1171/− 6.95%	2.4460/7.51%
Avg.	2.5189/10.97%	2.8419/0.00%	2.1917/22.86%	2.5898/8.66%	2.9406/4.74%

### Discussions

#### Variety of speed and transverse position

Precisely modeling the behaviors and distribution of vehicles on the bridge deck is crucial for the Traffic Load Models (TLMs). In this section, we analyze and discuss the variations in speed and transverse position of the actual-measured vehicles.

**Figure 12:**
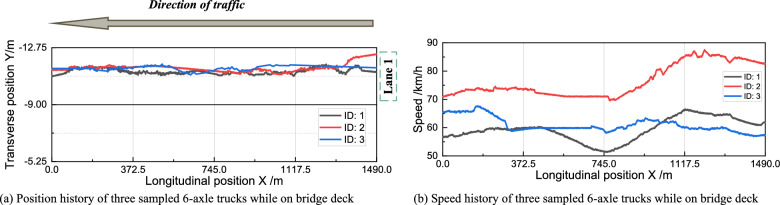
Actual-measured full-bridge trajectories of three 6-axle trucks.

Given that heavy vehicles significantly contribute to the fatigue damage of engineering components, we selected three samples of 6-axle trucks from Lane 1 as examples. Their positions and speeds on the deck are illustrated in Fig. 12. Overall, these vehicles exhibited varying speeds across the bridge deck, with speed fluctuations of up to 20 km/h. Additionally, their transverse positions showed short-range drift within their lanes. These observations align with previous studies in transportation engineering^27,28^.

As a result, *S-3* (i.e., the 2D-IDM model) reflects the actual movement characteristics of traffic loads with greater fidelity than other TLMs. Notably, *S-3* accounts not only for the variability in vehicle speeds but also for the statistical features of transverse movements.

#### Influence of gross weight threshold

The main contributors to suspender fatigue are typically heavy trucks with multiple axles. Consequently, excluding lighter vehicles from consideration can streamline the identification process and improve efficiency in the evaluation.

**Figure 13:**
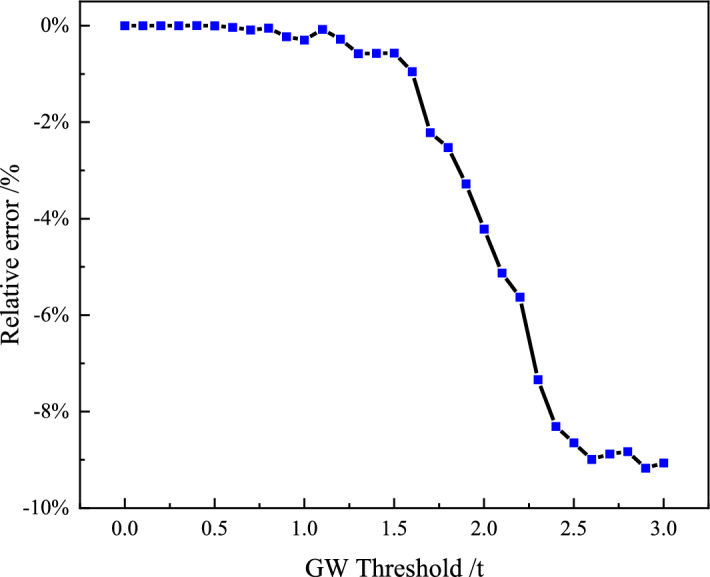
Relative errors of fatigue damage of S45 under GW thresholds.

To investigate the impact of lightweight vehicles, we established a range of successive gross weight (GW) thresholds, spanning from 0.0t to 3.0t. We used the benchmark model *O-2* to simulate spatial-temporal traffic loading, allowing us to eliminate vehicles weighing less than the specified threshold during the loading and damage calculation process for S45. The results are presented in Fig. 13.

Hence, in the context of this study on RSB, it is advisable not to disregard lightweight vehicles when conducting a detailed fatigue evaluation of a suspender. Nevertheless, it is worth noting that even though heavy-weight vehicles, representing only 22.4% of the total number, account for a minority, they contribute significantly, causing 90.94% of the fatigue damage for RSB.

#### Comprehensive comparison among different TLMs

A comprehensive summary and analysis of the characteristics, advantages, and shortcomings of different TLMs for fatigue evaluation are provided in Table 6. This table details the model type, data source, description, and traffic evolution parameters of these five TLMs. Furthermore, it offers an in-depth analysis of the advantages and disadvantages of each model.

**Table 6:** Comprehensive comparison among different TLMs.

TLM	Type	Data source	Description	Evolution param	Advantages	Disadvantages
*O-1*	Observation	WIM data	Actual collected data	–	Simple and efficient to apply	Can not consider the microscopic interactions between vehicles.High requirement of long-term relibility of WIM system.Post-evaluation of fatigue damage, not capable of prediction
*O-1*	Observation	WIM-Vision data fusion	Actual collected data	–	Precisely consistent to actual traffic loads	High requirement of long-term reliability of both WIM system and Machine Vision system (Fig. 5)Post-evaluation of fatigue damage, not capable of prediction
*S-1*	Simulation	WIM data	IDM simulator (with empirical param)	$v_0$, *T*, *a*, *b*, $s_0$	Only short-term WIM data is required	Not considerate with longitudinal and transverse motion features of actual traffic, causing large errors (Fig. 11, Table 5, 22.86% error in average)
*S-2*	Simulation	WIM data	IDM simulator (with calibrated param)	$v_0$, *T*, *a*, *b*, $s_0$	Only short-term WIM data is required. Capable of predictive fatigue damage evaluation with given time.Statistically aligned with traffic motion features at longitudinal direction.	Not considerate with transverse motion features of actual traffic, causing fatigue damage errors (Fig. 11, Table 5, 8.66% error in average)
*S-3*	Simulation	WIM-Vision data fusion	Proposed 2D-IDM simulator (with calibrated param)	$v_0$, *T*, *a*, *b*, $s_0$, $\mu _t$, $\sigma _t$	Only short-term WIM and Vision data is required.Capable of predictive fatigue damage evaluation with given time.Phenomenally (verified in Figs. 9, 10) and statistically aligned with traffic motion features at both longitudinal and transverse directions, causing fewer fatigue evaluation errors (Fig. 11, Table 5, 4.74% error in average)	Requirement of vehicle trajectory data, obtained by short-term WIM-Vision data fusion monitoring.More parameters to calibrate (Eq. 3, Table 4)

## Conclusions

To investigate the influence of vision-complemented Traffic Load Models (TLMs) on the fatigue evaluation of bridge suspenders, an experiment is conducted for a comparative study. Five typical TLMs are selected based on their data sources. Among them, *O-2* comprises traffic loads identified from WIM and Vision systems, serving as the benchmark model. *S-3* is introduced as a new 2D-IDM model for traffic load simulation. Driving characteristics learned from the full-span vehicle monitoring system are introduced into the improved IDM algorithm. Subsequently, the original FEM with beam-type main girder is refined and verified, to adapt to the precise surface loading procedure. Finally, the rainflow counting approach and Miner’s rule are applied to calculate the fatigue damage of the four shortest suspenders.

Detailed comparative analysis is implemented, results indicate the following: (1) Parameter calibration of a traffic simulator is deemed necessary for fatigue evaluation, as it exerts a substantial influence on the distribution and quantity of vehicles on the bridge deck. An average error of 22.86% is observed when the uncalibrated IDM (*S-1*) is used with its empirical parameters. (2) Models *O-1* and *S-2* solely rely on data from the WIM system. These models do not adequately account for the variability in speed and transverse position observed in practical measurements, resulting in average disparities of 10.97% and 8.66% for *O-1* and *S-2*, respectively. (3) The improved IDM model *S-3*, which involves the calibration of all parameters and incorporates a mechanism for transverse movement in the follower vehicle, outperforms other models in simulating practical traffic loads. An average error of only 4.74% is observed when comparing *S-3* with *O-2*. (4) The application of gross-weight thresholds for vehicles has a notable impact on the precision of fatigue evaluation. Based on the investigation, setting the threshold at 3.0t can result in a relative error of up to 9.06%. Thus, lightweight vehicles should not be disregarded in achieving high-fidelity fatigue evaluation for RSB in this study.

In the future, more complex fatigue calculation approaches will be explored, including the incorporation of vehicle–bridge interaction, considerations for corrosion, road roughness, and other relevant factors. Additionally, the models and parameters presented in this paper can be extended for the evaluation of various other bridge components.

## Data Availability

Data will be available on request by directly contacting the corresponding author at dongyiqing@tongji.edu.cn.
